# Association of Markers of Proinflammatory Phenotype and Beige Adipogenesis with Metabolic Syndrome in Chinese Centrally Obese Adults

**DOI:** 10.1155/2018/8956509

**Published:** 2018-02-18

**Authors:** Wilson K. C. Leung, Angus P. Yu, Christopher W. K. Lai, Parco M. Siu

**Affiliations:** ^1^Department of Health Technology and Informatics, The Hong Kong Polytechnic University, Hung Hom, Hong Kong; ^2^School of Public Health, Li Ka Shing Faculty of Medicine, The University of Hong Kong, Pokfulam, Hong Kong

## Abstract

**Background:**

Visceral adiposity is associated with higher productions of C-reactive protein (CRP) and interleukin-6 (IL-6). Inflammation of obese adipose tissues could contribute to systemic metabolic dysregulation, especially thermogenic activity of white adipose tissues, namely, beige adipogenesis, characterized by altered irisin expression. Thus, we investigated the roles of inflammation and adipocyte beiging in Chinese centrally obese (CO) adults with metabolic syndrome (MetS).

**Methods:**

This cross-sectional study was conducted on 54 CO and 58 non-CO subjects drawn from 1492 Chinese people with age and sex matched during November 2010 and August 2013. Twenty (37.0%) of the CO subjects fulfilled the IDF worldwide definition of MetS. Serum CRP, IL-6, and irisin levels were examined.

**Results:**

Higher CRP and IL-6, but lower irisin, levels were manifested in MetS versus non-MetS subjects with or without CO. Multiple linear regression identified high-density lipoprotein cholesterol level as the only independent risk factor for irisin level. Categorized by median of CRP and IL-6 levels, a lower irisin level was only observed in high CRP group.

**Conclusion:**

Under the condition of central obesity, chronic inflammation and impaired beige adipogenesis are associated with MetS in Chinese adults.

## 1. Introduction

People with metabolic syndrome (MetS) are at a heightened risk for overt diabetes and cardiovascular events [1]. Relative risks for atherosclerotic cardiovascular disease and type 2 diabetes within 5 to 10 years are approximately 2- and 5-fold in MetS versus non-MetS individuals, respectively [2]. Most individuals with MetS first acquire central (abdominal) obesity, and with aging and becoming more obese, multiple cardiovascular risk factors appear [3]. According to the International Diabetes Federation (IDF) worldwide definition, ethnicity-specific central obesity is a prerequisite risk factor for the diagnosis of MetS primarily based on its independent prognostic value for other clinical components of MetS and insulin resistance [4]. Obesity represents a pathophysiological condition largely characterized by chronic systemic inflammation in adipose tissues, particularly in regions of ectopic visceral fat deposition, causing release of proinflammatory cytokines which contribute to a spectrum of adverse clinical conditions from early signs of vascular endothelial dysfunction to atherosclerosis [5].

C-reactive protein (CRP), an established marker of inflammation, is an acute phase protein secreted from liver, playing a key role in the underlying causes of endothelial dysfunction as indicated by decreased nitric oxide and increased endothelin-1, as well as reduced plasminogen activator inhibitor-1 (PAI-1) levels [6]. Moreover, CRP also adversely correlated with the clinical features of MetS including triglycerides, fasting glucose and high-density lipoprotein cholesterol (HDL-C), and after age adjustment, there was a graded relationship between number of MetS diagnostic components and increased level of CRP [7]. Interleukin-6 (IL-6) is a pleiotropic cytokine or myokine with both pro- and anti-inflammatory properties [8]. It is active at sites of inflammation and therefore stimulates T- and B-cell maturation triggering chronic inflammatory responses [9]. High serum level of IL-6 was associated with higher risk of all-cause mortality in older women aged ≥ 65 years over a 3-year follow-up [10], as well as both all-cause and cardiovascular mortality in patients with coronary artery disease [11]. IL-6 was also strongly associated with arterial stiffness, markers of endothelial damage/dysfunction (e.g., von Willebrand factor), and deregulated coagulation and fibrinolytic systems (e.g., tissue factor, D-dimer, and tissue plasminogen activator) and conferred higher risk of having MetS [12, 13].

Adipose tissue, best known for energy storing in the form of fat, plays an important role in management of body weight and metabolic disorders through specialized, inducible transformation of preexisting, inguinal subcutaneous and visceral adipose tissues into thermogenic “brown-fat-like” adipocytes, also known as beige adipocytes [14]. In human and mouse obesity, dysfunctional beige adipogenesis is caused by inflammation of adipose tissues, and this inflammation-driven inhibition of adipocyte beiging has been proven to be self-sustained through signal circuitry between adipose tissue-resident macrophages and adipocytes currently [15]. Irisin is a newly identified, exercise-induced myokine acting on inguinal white adipose tissue depots through PPAR-*γ* coactivator-1 *α*/uncoupling protein-1 signaling cascades to elicit beige adipogenesis, muscular mitochondrial biogenesis, and fibre-type switching, resulting in loss of body weight [14, 16, 17]. It has been experimentally shown to improve fatty acid oxidation and glucose metabolism [18], delay atherosclerosis [19], and potentiate anti-inflammatory functions [20] *in vivo* and *in vitro* under normal or pathophysiological conditions. Serum irisin level, which has been significantly downregulated in type 2 diabetes patients [21–23], was associated with major adverse cardiovascular events in patients with established coronary artery disease after percutaneous coronary interventions [24], conferred with prognostic value for MetS and fasting plasma glucose in Chinese abdominally obese adults [25], and validated as an independent predictor for the severity of coronary artery disease in patients with stable angina [26].

In the present case-matched, cross-sectional study on Chinese centrally obese (CO) adults, we attempted to establish interrelationships of MetS and its individual diagnostic components with circulating levels of CRP, IL-6, and irisin and further substantiate the associations of proinflammation with the irisin levels. Completion of this study could confer a better understanding of inflammation-associated adipose complexity in obesity and hence potentially aid identification of therapeutic targets for cardiovascular complications.

## 2. Materials and Methods

### 2.1. Subjects

This case-matched, cross-sectional study was conducted on 112 subjects drawn from 1492 Hong Kong Chinese people aged from 24 to 86 years with age and sex matched during November 2010 and August 2013 [27, 28]. All subjects were volunteers recruited randomly from the community. The sampling strategy was based on age and sex, and 54 CO and 58 non-CO subjects were studied. Exclusion criteria included dementia, mental disorders, a medical history of cardiovascular diseases and stroke, neuromusculoskeletal disability or immobility, symptomatic pulmonary disorders, rheumatoid arthritis or osteoarthritis, cigarette or alcohol consumptions, and underlying metabolic disorders. Before the commencement of this study, all participants were fully informed about the purposes and procedures, and written informed consents were obtained. All subjects were identified with MetS according to the diagnostic guidelines of the IDF worldwide definition [4]. For an individual to be defined as having MetS, they must have central obesity (i.e., waist circumference ≥ 90 or ≥80 cm for Asian males and females, resp.) plus any two of the following clinical features:

1. Elevated blood pressure (BP) (i.e., systolic pressure ≥ 130 mmHg or diastolic pressure ≥ 85 mmHg)
2. Elevated plasma triglycerides [i.e., ≥1.70 mmol/L (or ≥150 mg/dL)]
3. Low level of HDL-C (i.e., ≤1.036 and ≤1.295 mmol/L for males and females, resp.)
4. Raised blood glucose [i.e., fasting glucose level ≥ 5.5 mmol/L (or ≥100 mg/dL)]

Waist circumference and blood pressure were measured by a trained researcher (Yu AP) [28]. Waist circumference of the subjects nearest to 0.1 cm was measured at the widest point of the waist, below the rib cage, and just above the hipbones or measured at the navel during exhalation. Blood pressure measurements were made using an electronic blood pressure monitor (Accutorr Plus, Datascope Inc., Montvale, New Jersey, USA) placed on the brachial artery while their arm was positioned at the heart level. Two measurements with 1-minute interval were taken, and the average value of the measurements was used for data analyses. Fasting blood specimens were collected and subsequently stored at −70°C for laboratory examinations. Fasting plasma glucose, triglyceride, and HDL-C levels were examined by an automated clinical chemistry analyzer (Architect CI8200, Abbott Diagnostics Park, IL) in a local accredited medical laboratory. The serum CRP (Abcam, Cambridge, MA), IL-6 (Abcam, Cambridge, MA), and irisin (BioVendor Laboratory Medicine, Brno, Czech Republic) levels were examined by commercially available ELISA kits according to the manufacturers' instructions. This study has been approved by the Human Subjects Ethics Subcommittee of the Hong Kong Polytechnic University (HSEARS20160810001).

### 2.2. Statistical Analysis

Data were presented as mean ± SD (standard deviation) or median (25th and 75th percentiles) for continuous variables and assessed for normality by Shapiro-Wilk test. Either one-way analysis of variance or Kruskal-Wallis test was used for comparing differences across MetS, CO, and non-CO groups. Group differences were compared using either Student's *t*- or Mann–Whitney *U* tests. Univariate and stepwise multiple linear regressions were employed to estimate the correlations of each clinical characteristic with serum CRP, IL-6, and irisin levels. A two-tailed test with *P* ≤ 0.05 was considered significantly different. All statistical analyses were conducted using SPSS Statistics 24.0.

## 3. Results

**Figure 1:**
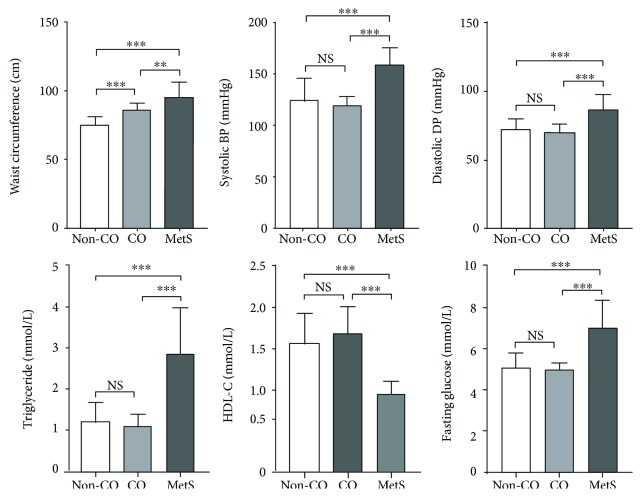
Clinical characteristics of Chinese subjects categorized by central obesity (CO) and metabolic syndrome (MetS) according to the IDF worldwide definition (*n* = 112). There were significantly higher waist circumference (^∗∗^*P* < 0.01 and ^∗∗∗^*P* < 0.001), systolic blood pressure (BP) (^∗∗∗^*P* < 0.001), diastolic BP (^∗∗∗^*P* < 0.001), plasma triglyceride (^∗∗∗^*P* < 0.001), and fasting blood glucose (^∗∗∗^*P* < 0.001), but lower high-density lipoprotein cholesterol (HDL-C) (^∗∗∗^*P* < 0.001) in MetS versus non-MetS subjects, whereas there was no significant difference between non-MetS subjects with or without CO, except waist circumference (^∗∗∗^*P* < 0.001).

The clinical characteristics of our subjects were categorized by the presence of CO in Table 1. Of the 54 CO subjects recruited, 20 subjects (37.0%) were identified with MetS. Therefore, there were 58 non-CO subjects, 34 CO subjects, and 20 CO with MetS subjects in the present study.  Figure 1 illustrated significantly higher waist circumference (*P* < 0.01 and *P* < 0.001), systolic BP (*P* < 0.001), diastolic BP (*P* < 0.001), plasma triglyceride (*P* < 0.001), and fasting blood glucose (*P* < 0.001), but lower HDL-C (*P* < 0.001) in MetS compared with non-MetS subjects with or without CO, respectively, whereas there was no significant difference between CO and non-CO, non-MetS subjects, except waist circumference (*P* < 0.001).

### 3.1. Serum Levels of CRP, IL-6, and Irisin Stratified by the Presence of MetS and CO

**Figure 2:**
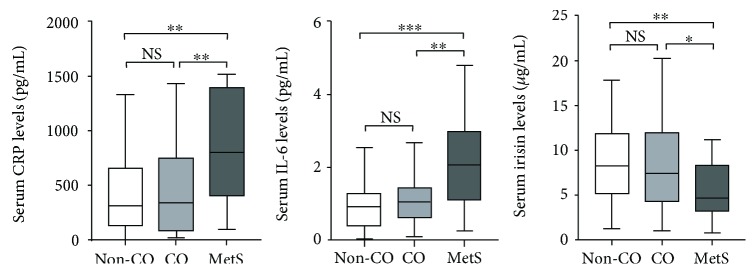
Serum levels of C-reactive protein (CRP), interleukin-6 (IL-6), and irisin of Chinese subjects categorized by central obesity (CO) and metabolic syndrome (MetS) according to the IDF worldwide definition (*n* = 112). There were significantly higher serum CRP (^∗∗^*P* < 0.01) and IL-6 levels (^∗∗^*P* < 0.01 and ^∗∗∗^*P* < 0.001), but lower irisin levels (^∗^*P* < 0.05 and ^∗∗^*P* < 0.01), in MetS versus non-MetS subjects. However, there was no significant difference in serum CRP, IL-6, and irisin levels between CO and non-CO, non-MetS cases.

There were significantly higher serum levels of CRP and IL-6 in MetS versus CO (*P* < 0.01) and non-CO, non-MetS subjects (*P* < 0.01) (Figure 2). On the other hand, lower irisin levels were manifested in MetS compared with CO (*P* < 0.05) and non-CO, non-MetS subjects (*P* < 0.01).

### 3.2. Correlations of Clinical Characteristics with Serum CRP, IL-6, and Irisin Levels

The correlations of clinical characteristics with serum levels of CRP, IL-6, and irisin were analyzed using univariate and stepwise multiple linear regression models. Serum CRP levels were shown to be positively correlated with systolic BP (*P* < 0.001), diastolic BP (*P* = 0.04), and triglycerides (*P* < 0.001), but negatively correlated with HDL-C levels (*P* < 0.001). Serum IL-6 levels were positively associated with waist circumference (*P* = 0.03), systolic BP (*P* = 0.008), diastolic BP (*P* = 0.04), triglycerides (*P* < 0.001), and fasting plasma glucose (*P* = 0.002) and also negatively correlated with HDL-C levels (*P* = 0.002). More importantly, serum irisin levels were negatively correlated with systolic BP (*P* = 0.04) and fasting plasma glucose (*P* = 0.03), but positively associated with HDL-C levels (*P* = 0.001) (Table 2). Stepwise multiple linear regression models identified HDL-C as the only independent prognostic risk factor for serum CRP (*P* < 0.001) and irisin (*P* = 0.005) levels, respectively, whereas systolic BP was found to be the only independent risk factor for serum IL-6 levels (*P* = 0.006).

### 3.3. Clinical Characteristics and Serum Irisin Levels Stratified by Median CRP and IL-6 Levels

In obesity, adipose tissue inflammation is associated with impaired inducible adipocyte beiging characterized by altered irisin levels [25, 26]. Subjects were therefore divided into two groups according to the median CRP [mean (±SD): 154.4 ± 111.1 and 839.5 ± 353.8 pg/mL, *P* < 0.001] and IL-6 [mean (±SD): 0.6 ± 0.3 and 3.1 ± 2.7 pg/mL, *P* < 0.001] levels (Tables 3 and 4). There were significantly higher systolic BP (*P* < 0.001) and triglycerides (*P* = 0.01), but lower HDL-C (*P* = 0.001) and irisin (*P* = 0.05) levels in high versus low CRP groups (Table 3). In high IL-6 group, significantly higher triglycerides (*P* = 0.003) and fasting plasma glucose (*P* = 0.009), but lower HDL-C (*P* = 0.02), were observed (Table 4).

## 4. Discussion

The present study revealed significantly higher circulating CRP and IL-6, but lower irisin, levels in MetS subjects. We also demonstrated an independent effect of HDL-C levels on CRP and irisin productions, whereas systolic BP was found to be the only independent predictor for IL-6 levels. Lower irisin levels were only clinically manifested in individuals with high CRP, but not IL-6, levels.

### 4.1. Proinflammation in Obesity and MetS

Welsh et al. [29] first established a direct molecular link between adiposity and inflammation using bidirectional Mendelian randomization approach on a randomized sample of 5804 elder patients aged ≥ 70 years with preexisting vascular disease (coronary, cerebral, or peripheral) or established risk factors of such disease (e.g., smoking, hypertension, or diabetes). Greater adiposity as indicated by body mass index-related single nucleotide polymorphisms was shown to confer higher circulating levels of CRP [29]. However, our findings were not completely in agreement with these previous works; the presence of central obesity in our non-MetS subjects could not lead to significantly elevate serum levels of CRP and IL-6. It could be explained by the fact that CRP and IL-6 are produced by a vast number of different cell types other than adipocytes under normal circumstances. Once beyond the specific cutoff for waist circumference to be defined as central obesity, multiple cardiovascular risk factors begin to appear [3, 4], and therefore, CRP and IL-6 were rapidly reaching remarkably high circulating levels in individuals with MetS. As supported by a cross-sectional survey on 1914 elder individuals aged 70–79 years without overt diabetes and cardiovascular events, there was a positively graded relationship between number of MetS diagnostic components and increased circulating levels of CRP and IL-6 [30].

### 4.2. Irisin in Obesity and MetS

Adipose tissue is metabolically active in nature and a highly secretory endocrine organ that is capable of modulating signals in appetite, energy expenditure, immune responses, inflammation, insulin sensitivity, and so forth [5]. In energy metabolism, irisin is specifically and highly expressed in inducible “brown-fat-like” adipocytes, also known as beige adipocytes, derived from inguinal white fat depots [14, 17]. Targeting circulating concentrations of irisin has been recently suggested to hold tremendous promise for treatment of metabolic disease since in irisin-treated normal or diet-induced type 2 diabetes mice, the activity of beige fat was selectively increased leading to suppressed weight gain, enhanced fatty acid oxidation, and improved glucose metabolism [14, 16, 18]. As shown in a similar study on Chinese middle-aged and older adults with CO and MetS, irisin levels were negatively associated with waist-to-hip ratio, and our findings were quite in line with this observation that albeit only tending to achieve statistical significance (*P* = 0.12), serum irisin levels were also negatively correlated with waist circumference (*R* = −0.15). In addition, stepwise multiple linear regression determined HDL-C as the only independent predictor for serum irisin levels, substantiating circulating irisin as a surrogate marker of improved lipid profile as previously described [31–33].

### 4.3. Irisin in MetS-Related Proinflammation

It is known that in obesity, inflamed adipose tissues could cause impaired beige adipogenesis and hence less efficient energy metabolism through complex interactions between macrophages and adipocytes [15], yet the underlying mechanism(s) remains poorly understood. This study is the first original research to report significantly lower serum irisin levels in Chinese adults with high CRP levels. CRP, a prototypic marker of inflammation, was clinically used to define proinflammatory phenotype in apparently healthy persons with consistently high and low CRP over a 1-year follow-up in terms of increased inflammation-related mediators including vascular cell adhesion protein 1, E-selectin, matrix metallopeptidase 9, and procoagulants such as PAI-1 and fibrinogen as well as lipopolysaccharide-induced whole blood TNF-0 and IL-1*β* levels [34]. In contrast, there was no significant difference in irisin levels between low and high IL-6 groups (*P* = 0.28). This phenomenon could be attributed to dual functions of IL-6 in both pro- and anti-inflammation, and its conflicting roles are largely dependent on (1) its expression levels of which lower extent exerts anti-inflammatory actions and vice versa, (2) different cell contexts as exemplified by its varying roles in hepatocytes, endothelial cells, skeletal muscle, and macrophages, and (3) its selective involvement in either classic or alternative transsignaling, where components of the transsignaling were positively associated with MetS, endothelial dysfunction, and arterial stiffness in male subjects at high risk of cardiovascular disease, and effective blockade of this signaling has been experimentally proven to attenuate innate immunity-triggered inflammatory responses in phenotypically IL-6-transsignaling knockout-like mice [8, 13]. However, it has to be kept in mind that this study is observational in nature, and there is no direct evidence of mechanistic insight linking CRP and IL-6 to irisin. Whether there is a signaling crosstalk among CRP, IL-6, and irisin deserves further investigation.

## 5. Conclusion

The present study provides a novel insight on how chronic inflammation affects the thermogenic activity of adipose tissue possibly through altered circulating levels of irisin in Chinese adults. Future studies are warranted to further delineate the exact mechanism(s) underlying the interplay among a spectrum of inflammatory mediators and irisin.

## Figures and Tables

**Table 1:** Clinical characteristics of Chinese subjects categorized by the presence of central obesity (*n* = 112).

	Central obesity (*n* = 54)	Noncentral obesity (*n* = 58)	*P* value
Age (years)	60.1 ± 9.9	60.4 ± 6.2	0.92
Waist circumference (cm)	90.6 ± 8.3	75.7 ± 5.6	<0.001
Systolic BP (mmHg)	135.1 ± 23.6	124.5 ± 21.4	0.001
Diastolic BP (mmHg)	76.5 ± 11.8	72.0 ± 8.0	0.04
Triglycerides (mmol/L)	1.8 ± 1.1	1.2 ± 0.5	<0.001
HDL-C (mmol/L)	1.3 ± 0.4	1.6 ± 0.4	<0.001
Fasting plasma glucose (mmol/L)	5.8 ± 1.3	5.1 ± 0.7	<0.001
CRP (pg/mL)^a,b^	484.9 (176.2, 933.2)	313.9 (125.4, 657.1)	0.024
IL-6 (pg/mL)^a,c^	1.1 (0.8, 2, 6)	0.9 (0.4, 1.3)	0.110
Irisin (*μ*g/mL)^a,b^	6.5 (3.5,1 0.8)	8.3 (5.1, 11.7)	0.055

^a^Statistical analyses carried out on available data. ^b^One outlier in CRP and Irisin were excluded. ^c^Two outliers in IL-6 was excluded. Data were mean ± SD for clinical characteristics, and median (25th, 75th) for serum CRP, IL-6, and irisin levels. BP: blood pressure; CRP: C-reactive protein; HDL-C: high-density lipoprotein cholesterol; IL-6: interleukin-6; MetS: metabolic syndrome.

**Table 2:** Linear regression of serum irisin level with clinical characteristics of Chinese subjects (*n* = 112).

	Univariate linear regression		Stepwise multiple linear regression^a^	
	Coefficient	*P* value	Coefficient	*P* value
Age (years)	−0.19	0.06	−0.09	0.38
Waist circumference (cm)	−0.15	0.12	—	—
Systolic BP (mmHg)	−0.20	0.04	−0.05	0.58
Diastolic BP (mmHg)	−0.04	0.71	—	—
Triglycerides (mmol/L)	−0.19	0.06	—	—
HDL-C (mmol/L)	0.31	0.001	0.27	0.005
Fasting plasma glucose (mmol/L)	−0.21	0.03	0.01	0.93

^a^Age and clinical characteristics significantly correlated with serum irisin level in univariate linear regression were further analyzed in multivariate model. BP: blood pressure; HDL-C: high-density lipoprotein cholesterol.

**Table 3:** Clinical characteristics of Chinese subjects categorized by serum CRP levels (*n* = 112).

	Low CRP^a^	High CRP^a^	*P* value
CRP (pg/mL)^b^	154.4 ± 111.1	839.5 ± 353.8	<0.001
Age (years)	59.7 ± 6.9	60.0 ± 8.8	0.77
Waist circumference (cm)	80.9 ± 8.8	83.7 ± 10.0	0.19
Systolic BP (mmHg)	121.0 ± 17.0	134.0 ± 8.2	<0.001
Diastolic BP (mmHg)	71.9 ± 23.3	75.2 ± 10.9	0.08
Triglycerides (mmol/L)	1.2 ± 0.5	1.7 ± 1.1	0.01
HDL-C (mmol/L)	1.6 ± 0.4	1.4 ± 0.4	0.001
Fasting plasma glucose (mmol/L)	5.2 ± 0.9	5.5 ± 1.2	0.38
Irisin (*μ*g/mL)^b,c^	8.5 (5.8, 11.7)	6.5 (3.6, 10.0)	0.05

^a^Serum CRP levels were categorized into low and high levels based on sample median. ^b^Statistical analyses carried out on available data. ^c^Outliers were excluded. Data were mean ± SD or median (25th, 75th) for serum irisin level. BP: blood pressure; CRP: C-reactive protein; HDL-C: high-density lipoprotein cholesterol.

**Table 4:** Clinical characteristics of Chinese subjects categorized by serum IL-6 levels (*n* = 112).

	Low IL-6^a^	High IL-6^a^	*P* value
IL-6 (pg/mL)^b^	0.6 ± 0.3	3.1 ± 2.7	<0.001
Age (years)	58.8 ± 7.9	60.4 ± 7.7	0.56
Waist circumference (cm)	80.6 ± 8.3	84.0 ± 11.9	0.36
Systolic BP (mmHg)	123.1 ± 17.1	134.1 ± 25.6	0.07
Diastolic BP (mmHg)	72.1 ± 9.0	75.9 ± 11.0	0.07
Triglycerides (mmol/L)	1.3 ± 0.7	1.7 ± 1.0	0.003
HDL-C (mmol/L)	1.6 ± 0.4	1.4 ± 0.4	0.02
Fasting plasma glucose (mmol/L)	5.2 ± 0.9	5.6 ± 1.1	0.009
Irisin (*μ*g/mL)^b,c^	7.4 (4.0, 10.8)	8.2 (5.1, 12.3)	0.28

^a^Serum IL-6 levels were categorized into low and high levels based on sample median. ^b^Statistical analyses carried out on available data. ^c^Outliers were excluded. Data were mean ± SD or median (25th, 75th) for serum irisin level. BP: blood pressure; HDL-C: high-density lipoprotein cholesterol; IL-6: interleukin-6.
